# Whole Genome Sequencing Allows Better Understanding of the Evolutionary History of *Leptospira interrogans* Serovar Hardjo

**DOI:** 10.1371/journal.pone.0159387

**Published:** 2016-07-21

**Authors:** Alejandro Llanes, Carlos Mario Restrepo, Sreekumari Rajeev

**Affiliations:** 1 Centro de Biología Celular y Molecular de Enfermedades, Instituto de Investigaciones Científicas y Servicios de Alta Tecnología (INDICASAT AIP), Ciudad del Saber, Panamá, Panamá; 2 Ross University School of Veterinary Medicine, Basseterre, St. Kitts & Nevis; University of Kentucky College of Medicine, UNITED STATES

## Abstract

The genome of a laboratory-adapted strain of *Leptospira interrogans* serovar Hardjo was sequenced and analyzed. Comparison of the sequenced genome with that recently published for a field isolate of the same serovar revealed relatively high sequence conservation at the nucleotide level, despite the different biological background of both samples. Conversely, comparison of both serovar Hardjo genomes with those of *L*. *borgpetersenii* serovar Hardjo showed extensive differences between the corresponding chromosomes, except for the region occupied by their *rfb* loci. Additionally, comparison of the serovar Hardjo genomes with those of different *L*. *interrogans* serovars allowed us to detect several genomic features that may confer an adaptive advantage to *L*. *interrogans* serovar Hardjo, including a possible integrated plasmid and an additional copy of a cluster encoding a membrane transport system known to be involved in drug resistance. A phylogenomic strategy was used to better understand the evolutionary position of the Hardjo serovar among *L*. *interrogans* serovars and other *Leptospira* species. The proposed phylogeny supports the hypothesis that the presence of similar *rfb* loci in two different species may be the result of a lateral gene transfer event.

## Introduction

Leptospirosis is a bacterial zoonosis which impacts both human and animal health worldwide and is caused by pathogenic members of the genus *Leptospira*. The members of this genus have been classified into more than 250 serovars, grouped into 24 antigenically related serogroups [1]. This classification is based on serovar-specific antisera reacting mainly against components of the surface lipopolysaccharide (LPS). DNA-DNA hybridization has been used to classify *Leptospira* into species, while phylogenetic studies based mainly on 16S rRNA sequences have been used to further classify species into groups [2]. However, correlation between serological and DNA-based classification is poor, as members of the same serovar may belong to different species [3].

Some *Leptospira* serovars are adapted to a particular host species and asymptomatically infect renal tubules during host adaptation. Certain serovar determinants, such as the O-antigen of LPS, are thought to be involved in host selection by mechanisms that are largely unknown [4]. Serovar Hardjo belonging to *L*. *interrogans* and *L*. *borgpetersenii* are known to be adapted to cattle. *L*. *borgpetersenii* serovar Hardjo is the most common host-adapted species in cattle all over the world, while the pattern of host adaptation for *L*. *interrogans* serovar Hardjo is relatively unclear [5]. Although both belong to two different species, they were confirmed to have a very similar *rfb* locus, the gene cluster encoding the enzymes involved in LPS biosynthesis [6]. The presence of almost identical *rfb* loci can be associated with a similar LPS structure, which further explains the similar serological reaction in these rather different species. A convergent evolution of the loci due to acquisition of genes via lateral gene transfer was proposed as an explanation for this observation.

*L*. *interrogans* serovar Hardjo differs from its *L*. *borgpetersenii* counterpart in several clinical and epidemiological aspects. Infection of cattle with *L*. *interrogans* serovar Hardjo has been associated with a rate of abortion of 30%, reasonably higher than that caused by *L*. *borgpetersenii* serovar Hardjo, which is around 3–10% [7]. In addition, *L*. *interrogans* serovar Hardjo has been specifically associated with the development of milk drop syndrome in dairy cows as a consequence of acute infection [8]. The genome of a field isolate of *L*. *interrogans* serovar Hardjo (strain Norma) was recently published [9], however no functional or evolutionary analysis was provided in the article. In the present study, we sequenced, assembled and annotated the genome of a laboratory-adapted strain of *L*. *interrogans* serovar Hardjo (serogroup Sejroe, strain Hardjoprajitno). Analysis of the sequenced chromosomes allowed us to further study the genetic background of *L*. *interrogans* serovar Hardjo and its evolutionary relationship with other members of the genus *Leptospira*.

## Materials and Methods

### Bacterial Culture and DNA Extraction

The *L*. *interrogans* serovar Hardjo strain sequenced in this study was obtained from the National Veterinary Services Laboratory (Ames, Iowa, USA). This strain was originally isolated from a human patient in Indonesia and has been routinely used in veterinary diagnostic laboratories in the United States. The strain was maintained in the laboratory in Ellinghausen-McCullough-Johnson-Harris (EMJH) medium and passaged for several generations. Genomic DNA for sequencing was isolated using the MasterPure^™^ Complete DNA and RNA Purification Kit (Epicentre, USA) following manufacturer’s instructions.

### Genome Sequencing, Assembly and Annotation

Genomic DNA was sequenced at the Georgia Genomics Facility of the University of Georgia (UGA) by using the Illumina MiSeq technology with standard protocols. Reads were trimmed and cropped to 250 bp by using trimmomatic [10] to remove low-quality positions from the 3' end. *De novo* assembly was performed by using SPAdes [11], with the recommended options for MiSeq reads. Contigs were further scaffolded by using ABACAS [12] and the genome of *L*. *interrogans* serovar Lai strain 56601 [13] as a reference. Gene models annotated in the genome of serovar Lai and in that of the closely related serovar Copenhageni strain Fiocruz L1-130 [14] were transferred to the contiguated pseudochromosomes by using RATT [15]. BaSYS [16] was also used for *de novo* gene detection. All lines of evidence for gene models were manually revised and merged using Artemis and the Artemis Comparison Tool (ACT) [17]. To avoid the relatively high levels of over-annotation that has been reported for other *Leptospira* genomes when using automatic pipelines [18], we followed the guidelines described by Bulach *et al*. [19] for the annotation of two *L*. *borgpetersenii* serovar Hardjo genomes. Read mapping to reference genomes was performed with BWA [20] and variant calling from read alignments was done with SAMtools (v. 0.1.19) [21].

### Functional and Phylogenomic Analyses

OrthoMCL [22] was used to cluster the genes of the newly annotated serovar Hardjo genome along with those from another 23 selected *Leptospira* genomes previously submitted to Genbank (Table 1). Each ortholog group was tested for evidence of selection, either positive or negative, by comparing models 1 and 2 of the codeml program from the PAML 4 package [23]. Protein sequences were aligned with MAFFT [24] and the alignments were further refined by using Gblocks [25]. Phylogenetic trees were built with PhyML 3.0 [26] under the best model predicted by ProtTest3 [27], with bootstrap values for branch support resulting from 500 bootstrap replicates.

**Table 1:** Genomes of *Leptospira* species, strains and serovars selected for the functional and phylogenomic studies presented in this article.

Species	Serovar	Strain	Locus tag prefix	NCBI BioProject
*L*. *biflexa*	Patoc	Patoc 1 (Ames)	LBF	PRJNA20133
*L*. *borgpetersenii*	Hardjo (type A)	L550	LBL	PRJNA16146
	Hardjo (type B)	JB197	LBJ	PRJNA16148
	Mini	200901116	LEP1GSC190	PRJNA167259
	Pomona	200901868	LEP1GSC133	PRJNA167255
*L*. *interrogans*	---	Brem 329	LEP1GSC057	PRJNA167229
	---	FPW1039	LEP1GSC079	PRJNA167242
	---	FPW2026	LEP1GSC080	PRJNA74077
	Bataviae	L1111	LEP1GSC087	PRJNA74089
	Bulgarica	Mallika	LEP1GSC007	PRJNA65041
	Canicola	Fiocruz LV133	LEP1GSC069	PRJNA167235
	Copenhageni	Fiocruz L1-130	LIC	PRJNA10687
	Lai	56601	LA	PRJNA293
	Lai	IPAV	LIF	PRJNA32553
	Linhai	56609	LIL	PRJNA217894
	Hardjo	Norma	G436	PRJNA185511
	Manilae	UP-MMC-NIID LP	LIMLP	PRJNA287300
	Pomona	Pomona	LEP1GSC014	PRJNA65043
	Pyrogenes	2006006960	LEP1GSC019	PRJNA74039
*L*. *kirschneri*	---	H1	LEP1GSC081	PRJNA74079
	---	H2	LEP1GSC082	PRJNA167243
*L*. *licerasiae*	Varillal	VAR 010	LEP1GSC185	PRJNA74167
*L*. *santarosai*	Shermani	LT 821	LSS	PRJNA47139
*L*. *weilii*	---	UI 13098	LEP1GSC108	PRJNA74123

## Results

### Assembly and Annotation of the *L*. *interrogans* Serovar Hardjo Genome

Raw sequences of MiSeq reads generated in this study were deposited in the Sequence Read Archive (SRA) under the accession code SRX1830060. *De novo* assembly of the MiSeq reads with SPAdes resulted in 101 contigs larger than 500 bp, with an N50 size of 168 kb and a total size of 4.76 Mb. We were able to contiguate 71 of these contigs into two pseudomolecules corresponding to chromosomes I (4.34 Mb) and II (353 kb), by using ABACAS and the genome of *L*. *interrogans* serovar Lai as a reference. The remaining 30 contigs could not be incorporated into the contiguated pseudomolecules mainly due to their repetitive nature. This set of unplaced contigs represents a 2% of the total bases in the original *de novo* assembly and are available to download from our project’s website (http://bioinfo.indicasat.org.pa/lepto.html).

RATT was able to transfer 97% and 98% of the gene models annotated in the genomes of *L*. *interrogans* serovar Lai and Copenhageni, respectively, to the contiguated pseudochromosomes. The transferred gene models were manually revised and combined with 4,739 additional ones predicted by BaSYS. Final revision of the annotation included 3,754 predicted protein-coding genes, 3,466 in chromosome I and 288 in chromosome II. We also annotated 86 suspected pseudogenes, identified on the basis of models transferred by RATT with at least one frameshift or internal stop codon, in cases where those artifacts could be confirmed in the majority of the corresponding reads. The annotated genome was deposited in GenBank under BioProject PRJNA296687 with accession numbers CP013147 (chromosome I) and CP013147 (chromosome II). General statistics regarding size and gene content of our annotated genome are roughly similar to those of serovars Lai and Copenhageni (Table 2).

**Table 2:** Basic statistics of the *L*. *interrogans* serovar Hardjo genome sequenced in this study, compared with those of *L*. *interrogans* serovar Lai (strain 56601), *L*. *interrogans* serovar Copenhageni (strain Fiocruz L1-130) and *L*. *interrogans* serovar Hardjo (strain Norma).

Feature	*L*. *interrogans* sv. Lai str. 56601	*L*. *interrogans* sv. Copenhageni str. Fiocruz L1-130	*L*. *interrogans* sv. Hardjo str. Norma	*L*. *interrogans* sv. Hardjo str. Hardjoprajitno
Chromosomes	2	2	2	2
Chr I size (Mb)	4.34	4.28	4.41	4.34
Chr II size (Mb)	0.36	0.35	0.36	0.35
Total size (Mb)	4.70	4.63	4.77	4.69
GC-content (%)	35.02	35.04	35.02	35.00
Protein-coding genes	3,683	3,793	4,696	3,754
Transfer RNA	37	37	37	37
Ribosomal RNA	5	5	5	3

Our genome is very similar to that recently published for a field isolate of *L*. *interrogans* serovar Hardjo (strain Norma) [9]. Regions that could be aligned on a one-to-one basis between both genomes share a 99.9% identity at the nucleotide level. These regions in turn represent a 98.4% of the strain Norma genome, with the remaining 1.6% comprising approximately 70 kb of sequence that could not be found in our assembly, 67 kb from chromosome I and 2 kb from chromosome II. These sequences roughly match to most of the contigs we were not able to contiguate into our assembled pseudochromosomes due to their repetitive nature. All of these contigs contain matches to sequences associated with repetitive elements commonly reported to be present in *Leptospira* genomes [4], including prophages, transposons and insertion sequence (IS) elements. Assembly of such elements is difficult, especially when using relatively short reads such as those generated by using the MiSeq platform. We assume that such sequences could be unequivocally incorporated into the strain Norma assembly because of to the larger size of reads generated by the Roche’s 454 platform. To better study sequence variation between the genomes of both strains, we aligned the raw reads from strain Hardjoprajitno to the strain Norma genome. As expected, 99.8% of the reads from strain Hardjoprajitno could be unambiguously mapped to the strain Norma genome, with a uniform coverage along both chromosomes (S1 Fig). We found 262 single nucleotide polymorphism (SNPs) located within predicted protein-coding genes between both genomes (S1 Table). This number of SNPs is relatively low when compared to those found when mapping the strain Hardjoprajitno reads to the reference genomes of *L*. *interrogans* serovars Lai and Copenhageni, which were in the order of 15,000 and 16,000, respectively (results not shown).

**Fig 1:**
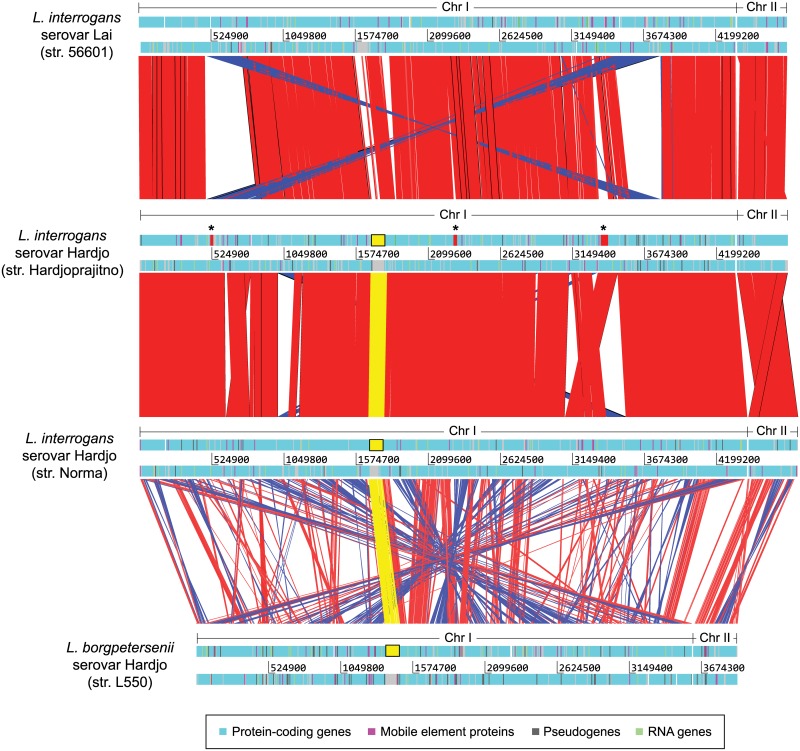
Comparison of the two *L*. *interrogans* serovar Hardjo genomes included in this study with those of *L*. *interrogans* serovar Lai (strain 56601) and *L*. *borgpetersenii* serovar Hardjo (strain L550). Red bands indicate similar regions and blue bands indicate inversions. Sequences corresponding to the *rfb* loci typical of the Hardjo serovar are highlighted in yellow. Three regions present in the *L*. *interrogans* serovar Hardjo genome but absent from that of the Lai serovar are indicated by asterisks.

Except for those resulting from unplaced contigs, we found relatively few structural differences between the corresponding chromosomes from strains Norma and Hardjoprajitno (Fig 1, S2 Table). However, the number of predicted gene models is surprisingly higher in the strain Norma genome when compared to our annotation or to those of serovars Lai and Copenhageni. This unexpected level of over-annotation in the strain Norma genome is likely to be a consequence of using an automatic pipeline, a situation that has been reported for other *Leptospira* genomes previously sequenced [18]. Although over-annotation complicates the comparison at the level of gene content, we found no differences among those predicted genes and pseudogenes that are shared by both strains.

### Comparative Genomics of the Hardjo Serovar

Comparison of the *L*. *interrogans* serovar Hardjo genome with those of serovars Lai and Copenhageni revealed relatively high sequence similarity, except for the region occupied by their corresponding *rfb* loci (Fig 1, S2 Fig). We noticed two inverted transpositions in the genomes of serovar Hardjo when compared to the genome of serovar Lai, which are located near the ends of the larger inversion previously reported between the genomes of serovar Lai and Copenhageni. In contrast, comparison of the *L*. *interrogans* serovar Hardjo genomes with those of *L*. *borgpetersenii* serovar Hardjo type A and type B [19] revealed extensive sequence and structural variation, but high conservation in the region of the *rfb* locus.

We also observed three regions in chromosome I of both serovar Hardjo strains that are not apparently present in the genomes of serovars Lai and Copenhageni (Fig 1, S3 Table). The first of these regions has an approximate length of 12 kb and includes 18 predicted genes (LIH_02395-LIH_02480 in strain Hardjoprajitno). The only gene in the region whose function could be predicted was one located near its beginning, putatively encoding an ISX02-like transposase.

The second region is slightly larger (~17 kb) and encompasses 27 predicted genes (LIH_09760-LIH_09890 in strain Hardjoprajitno). All these genes are encoded in the same strand and are very close together, which suggests that they may be co-transcribed as an operon. Again, functions could not be predicted for most genes in the region, except for two that appear to encode peptidases (LIH_09765 and LIH_09765) and two adjacent ones that appear to encode a PIN domain protein (LIH_09845) and a plasmid replication initiation factor (LIH_09850), respectively. The PIN domain is typically found in ribonucleases that act as the toxin component of type II toxin-antitoxin systems (TAs) [28]. Despite its relatively high sequence similarity to those described in TAs, the PIN domain protein described here does not appear to be adjacent to a gene encoding a putative antitoxin component, suggesting that if active, it may fulfill a different function.

The third region is the largest one, spanning ~37 kb and including 30 predicted genes (LIH_14055-LIH_14190 in strain Hardjoprajitno). Notably, the region contains a cluster of genes predicted to encode components of a transporter from the **r**esistance-**n**odulation-cell **d**ivision (RND) superfamily. Although several types of RND transporters have been described, the one we report here seems to be of the tripartite type. This type is the most common one in Gram-negative bacteria and is composed of an inner membrane exporter protein (AcrB), a periplasmic membrane fusion protein (AcrA or MFP) and an outer membrane channel protein (TolC) [29]. The cluster we found in this region includes two genes predicted to encode AcrB transporters (LIH_14115 and LIH_14120), directly adjacent to a gene encoding the AcrA subunit (LIH_14125) and a close gene encoding a TolC-like outer membrane efflux protein (LIH_14150). A similarity search with the sequences of these genes revealed the presence of a similar cluster in the genomes of most pathogenic species of *Leptospira* other than *L*. *interrogans*. For *L*. *interrogans*, a similar cluster could only be detected in serovars Bataviae, Canicola and Pyrogenes.

Similarly, genes from the other two previously described regions appear to be present only in some *L*. *interrogans* serovars and other close *Leptospira* species. Genes from the first region appear to be present only in *L*. *interrogans* serovar Hardjo, while those from the second region are present in *L*. *santarosai*, *L*. *kirschneri* and *L*. *weilii*, with only a few of them having detectable orthologs in serovars Bataviae and Manilae of *L*. *interrogans*.

We were also able to detect orthologs for many of these genes in genomes of unidentified serovars, submitted to Genbank as part of the *Leptospira* Genomics and Human Health Project sponsored by the J. Craig Venter Institute. Remarkably, almost all of these genes have collinear orthologs in the genome of a strain Brem 329 isolated from a horse in Germany (BioProject PRJNA167229). We also found a 99.9% identity at the nucleotide level between this genome and the serovar Hardjo genome sequenced in this study. The corresponding *rfb* loci of both genomes are also very similar. These findings suggest that the Brem 329 strain may belong to the Hardjo serovar. Similarly, several loci in these regions have detectable orthologs in the genomes of strains FPW1039 (BioProject PRJNA167242) and FPW2026 (BioProject PRJNA74077). However, these genomes share lower sequence similarity with that of serovar Hardjo, both overall and in the *rfb* loci, which suggests that the strains may belong to different but probably evolutionarily related serovars.

### Phylogenomic Approach to Study the Evolutionary Position of the Hardjo Serovar

Given the differences observed among serovar Hardjo and other *L*. *interrogans* serovars, their relationship in the context of the evolutionary history of *Leptospira* species was explored. We utilized a strategy based on concatenation of sequences from orthologous genes, since commonly used phylogenetic markers such as 16S rRNA do not provide enough phylogenetic signal to properly separate serovars of the same species in phylogenies [30,31]. In this strategy, the phylogenetic signal is increased by including as many genes as possible from the sequenced genomes.

We initially performed an ortholog clustering analysis with the gene models annotated in the *L*. *interrogans* serovar Hardjo genome along with those of 23 selected *Leptospira* specimens. This set of genomes was selected on the basis of the differences we observed and mentioned in the previous section, and it included one strain of *L*. *biflexa*, *L*. *licerasiae* and *L*. *santarosai*, four strains of *L*. *borgpetersenii* (two of them from the Hardjo serovar), two strains of *L*. *kirschneri* and 12 strains of *L*. *interrogans*. Nine of these *L*. *interrogans* strains belong to identified serovars, two of serovar Lai and one of serovars Canicola, Bataviae, Pyrogenes, Linhai, Manilae and Pomona, respectively. The remaining three strains included in the study are those with unidentified serovar mentioned in the previous section. This analysis resulted in 8,470 ortholog groups, out of which 1,565 have only one representative member in all the genomes considered (S4 Table).

**Fig 2:**
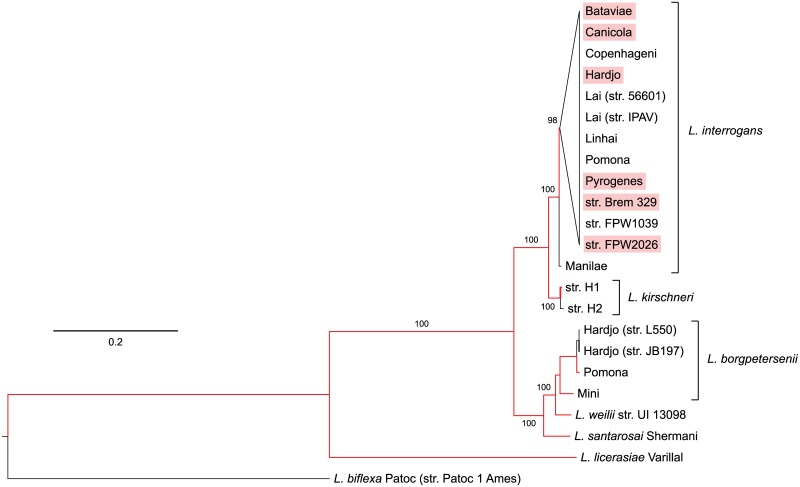
Maximum likelihood tree based on concatenated protein sequences from 23 *Leptospira* genomes. The saprophytic non-pathogenic *L*. *biflexa* was used to root the tree. Branches highlighted in red are those leading to taxa whose genomes contain the additional cluster of RND transporter components described for serovar Hardjo (see main text). As there was not enough phylogenetic signal to separate individual *L*. *interrogans* serovars, those having this cluster are shaded in pink. Bootstrap values are shown for branches separating different species.

Although, as an initial approach we planned to include all of these “core” genes in the phylogenomic analysis, it has been shown that concatenating sequences from genes subjected to different evolutionary pressures may lead to erroneous phylogenetic reconstructions [32]. To avoid this, we looked for evidence of natural selection in all ortholog groups and further selected the groups suspected to have a neutral or nearly neutral evolution, that is, those with an overall dN/dS ratio between 0.2–2.0, as suggested by Massey *et al*. [32]. Of the 1,565 “core” genes, only 235 meet this criterion. Alignment of the concatenated amino acid sequences of those genes with MAFFT and further refinement with Gblock yielded an alignment of ~53,500 sites. A maximum likelihood tree based on this alignment was built by using PhyML and the LG+G+I model (Fig 2).

**Fig 3:**
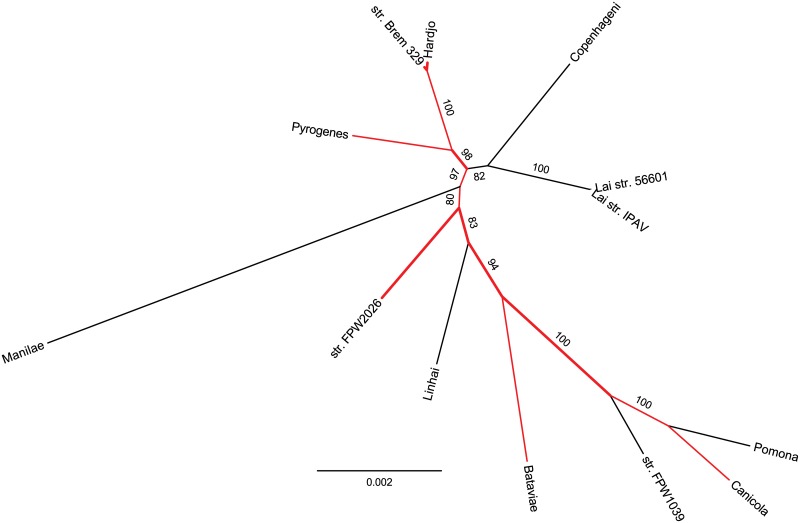
Unrooted tree of selected *L*. *interrogans* serovars. This maximum likelihood tree was built following the same methodology described for the tree in Fig 2, but considering only the *L*. *interrogans* serovars. Branches highlighted in red are those corresponding to serovars whose genomes contain the additional cluster of RND transporter components. Bootstrap values are shown for branches separating different serovars.

The topology of this tree agrees with the widely accepted phylogeny for the *Leptospira* genus [4]. However, individual *L*. *interrogans* serovars could not be properly separated by using only this set of genes, as there is still not enough phylogenetic signal. In an attempt to increase the signal, we repeated this analysis only on *L*. *interrogans* serovars, where the number of orthologs matching the selection criterion increased to 512. The new maximum likelihood tree was built with an alignment of ~121,000 concatenated sites (Fig 3).

Both trees show that Manilae is likely to be the serovar closest to the ancestral position within the *L*. *interrogans* clade. Suggestion of the relatedness between *L*. *interrogans* serovar Hardjo and the Brem 329 strain of unidentified serovar is supported by their position in the tree, which is similar to that observed for strains 56601 and IPAV, both of which belong to the same Lai serovar. The topology also shows that serovar Pyrogenes seems to be more closely related to Hardjo. Strain FPW2026 is positioned between serovars Manilae and Linhai, and strain FPW1039 is closer to serovars Pomona and Canicola.

## Discussion

Whole genome sequencing allowed us to study the evolutionary relationship of *L*. *interrogans* serovar Hardjo with different serovars of *L*. *interrogans* and other species of the *Leptospira* genus. The suggested evolutionary position of serovar Hardjo supports the hypothesis that the convergence of the *rfb* loci from *L*. *interrogans* serovar Hardjo and *L*. *borgpetersenii* serovar Hardjo are likely to be the consequence of an ancestral lateral gene transfer event, as both are grouped in separate clades corresponding to their respective species.

Comparison of the genomes of *L*. *interrogans* serovar Hardjo strain Hardjoprajitno and *L*. *interrogans* serovar Hardjo strain Norma revealed relatively high sequence conservation, despite the fact that these strains have very different origins, the first one is a laboratory-adapted strain sampled from a male patient from Indonesia many years ago, while the second one is a field isolate recently sampled from infected cattle in Brazil. We found a few structural rearrangements between the corresponding chromosomes of both strains, which does not appear to affect protein-coding genes, except for some of them predicted to code for mobile element proteins. In fact, most of these rearrangements appear to be flanked by genes predicted to encode transposases, suggesting that the corresponding mobile elements may have played a role in their transposition. It is important to mention, however, that these rearrangements may ultimately be the result of assembly errors, as sequencing was in both cases performed by using next-generation sequencing techniques and no PCR or other type of experimental validation was conducted.

Comparisons of the sequenced *L*. *interrogans* serovar Hardjo genomes and those of other *Leptospira* species also allowed us to identify three relatively large regions present in this serovar, with a limited distribution among other *Leptospira* genomes. Among these regions, the one containing the gene encoding a PIN domain protein may be reminiscent of an integrated plasmid, as such proteins and their associated TA operons are commonly found in plasmids and are thought to play a role in plasmid stability [28]. Furthermore, the region also contains a gene putatively encoding a plasmid replication initiation factor. The loss of the region from chromosome I of several other *L*. *interrogans* serovars may be explained by a large deletion event or by its re-excision from the chromosome into a plasmid, a phenomenon that has been previously described in the species [33].

Another region was found to contain an additional cluster of genes putatively encoding components of a tripartite RND transporter system. Like most Gram-negative bacteria, *Leptospira* has several genes encoding transporters of the RND superfamily. For example, of all the genes encoding putative membrane transporters in *L*. *interrogans* serovar Copenhageni, 11% appear to be related to the RND superfamily [14]. In the genome of *L*. *interrogans* serovar Lai there are at least 14 loci predicted to encode the inner membrane exporter protein AcrB, although only two of these loci have a structure similar to the additional cluster we described for serovar Hardjo. This particular type is composed of one or two acrB genes, which appear to form an operon with an acrA gene and a nearby gene encoding a TolC-like protein. Phylogenetic analysis shows that the genes for this additional cluster may have been acquired by an ancestor close to *L*. *licerasiae* and subsequently lost in some lineages (Fig 2). A phylogenetic tree built with the sequences of the acrA paralogs confirmed the presence of three clearly different lineages for this gene among *Leptospira* genomes (S3 Fig). It has been shown that RND transporters and especially their increased number of copies are actively involved in the development of drug resistance [34]. Although this analysis is preliminary, the additional copy of this cluster may represent an adaptive advantage in those lineages that have maintained the copy during their evolution.

Although functions could not be predicted for the vast majority of genes in these regions, it is likely that many of them are involved in differences in pathogenicity reported for *L*. *interrogans* serovar Hardjo and should be the target of future experimental research.

## Supporting Information

S1 Fig Mapping of the reads from *L*. *interrogans* serovar Hardjo strain Hardjoprajitno to the genome of *L*. *interrogans* serovar Hardjo strain Norma. Raw read depth plotted in light blue was averaged over a window of 500 bp. Vertical red bars below the coverage plot indicate SNPs located within predicted protein-coding genes.
(PDF)

S2 Fig Comparison of the two *L*. *interrogans* serovar Hardjo genomes included in this study with those of serovars Lai and Copenhageni. Red bands indicate similar regions and blue bands indicate inversions. Sequences corresponding to the *rfb* loci typical of the Hardjo serovar are highlighted in yellow.
(PDF)

S3 Fig Maximum likelihood tree of acrA genes. This tree was built with the amino acid sequences of the acrA genes from the RND transporter gene clusters present in the *Leptospira* genomes used in this study. The tree was built with PhyML 3.0 using the LG model and 500 bootstrap replicates. Bootstrap values are indicated for branches clustering the genes from different species. Genes from the strain sequenced in this study are indicated in bold.
(PDF)

S1 Table Single nucleotide polymorphisms (SNP) within predicted protein-coding genes between the genomes of *L*. *interrogans* serovar Hardjo strains Hardjoprajitno and Norma. (XLSX)

S2 Table Structural rearrangements between corresponding chromosomes from the genomes of *L*. *interrogans* serovar Hardjo strain Norma and *L*. *interrogans* serovar Hardjo strain Hardjoprajitno. (XLSX)

S3 Table Genes that are present in *L*. *interrogans* serovar Hardjo but not present in serovars Lai or Copenhageni. (XLSX)

S4 Table Ortholog groups used in phylogenomic analysis for species and serovars. (XLSX)
