# Supplementary material for: Whole Genome Sequencing Allows Better Understanding of the Evolutionary History of Leptospira interrogans Serovar Hardjo
Source: PLoS One. 2016 Jul 21;11(7):e0159387. doi: 10.1371/journal.pone.0159387 (PMC4956267; doi:10.1371/journal.pone.0159387)
Supplement: S1 Fig — Raw read depth plotted in light blue was averaged over a window of 500 bp. Vertical red bars below the coverage plot indicate SNPs located within predicted protein-coding genes. (PDF) [file pone.0159387.s001.pdf]

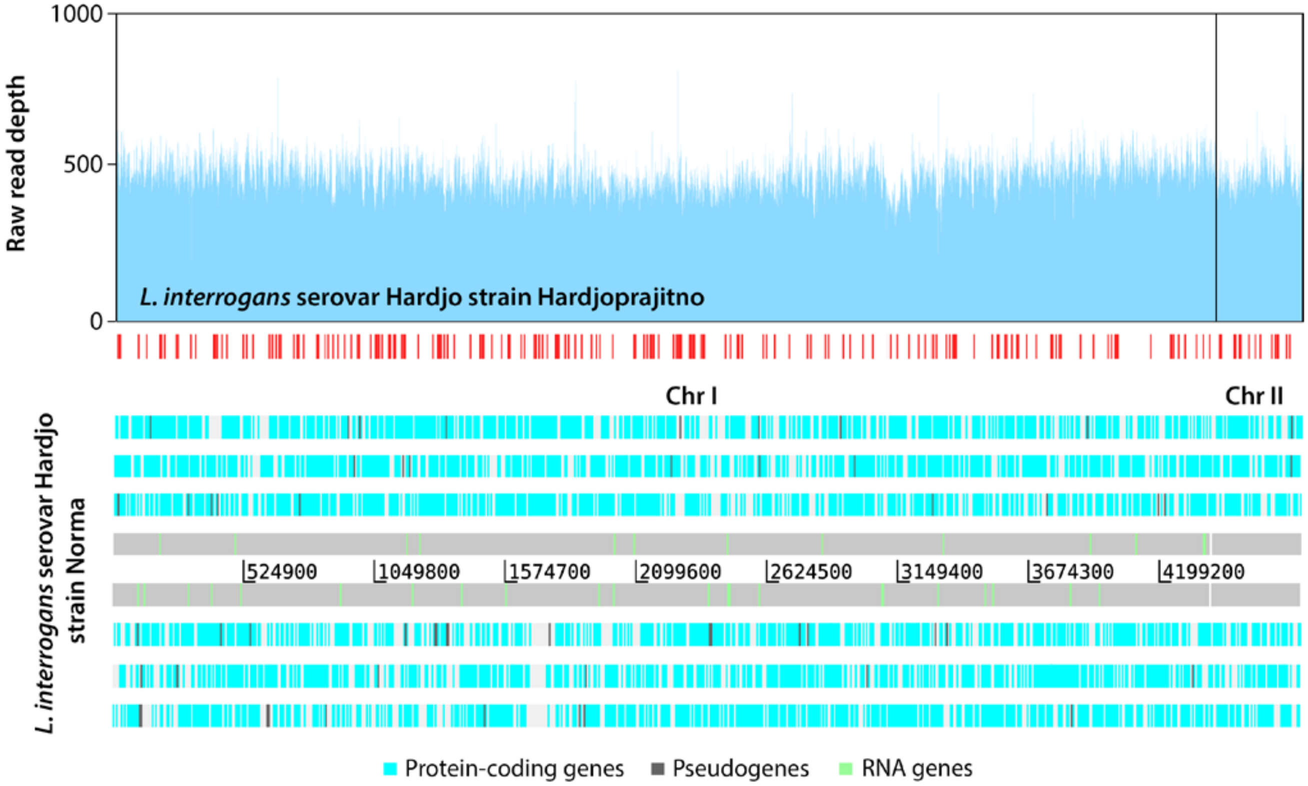

Figure S1. Mapping of the reads from *L. interrogans* serovar Hardjo strain Hardjoprajitno to the genome of *L. interrogans* serovar Hardjo strain Norma. Raw read depth plotted in light blue was averaged over a window of 500 bp. Vertical red bars below the coverage plot indicate SNPs located within predicted protein-coding genes.
