# Supplementary material for: Whole Genome Sequencing Allows Better Understanding of the Evolutionary History of Leptospira interrogans Serovar Hardjo
Source: PLoS One. 2016 Jul 21;11(7):e0159387. doi: 10.1371/journal.pone.0159387 (PMC4956267; doi:10.1371/journal.pone.0159387)
Supplement: S2 Fig — Red bands indicate similar regions and blue bands indicate inversions. Sequences corresponding to the rfb loci typical of the Hardjo serovar are highlighted in yellow. (PDF) [file pone.0159387.s002.pdf]

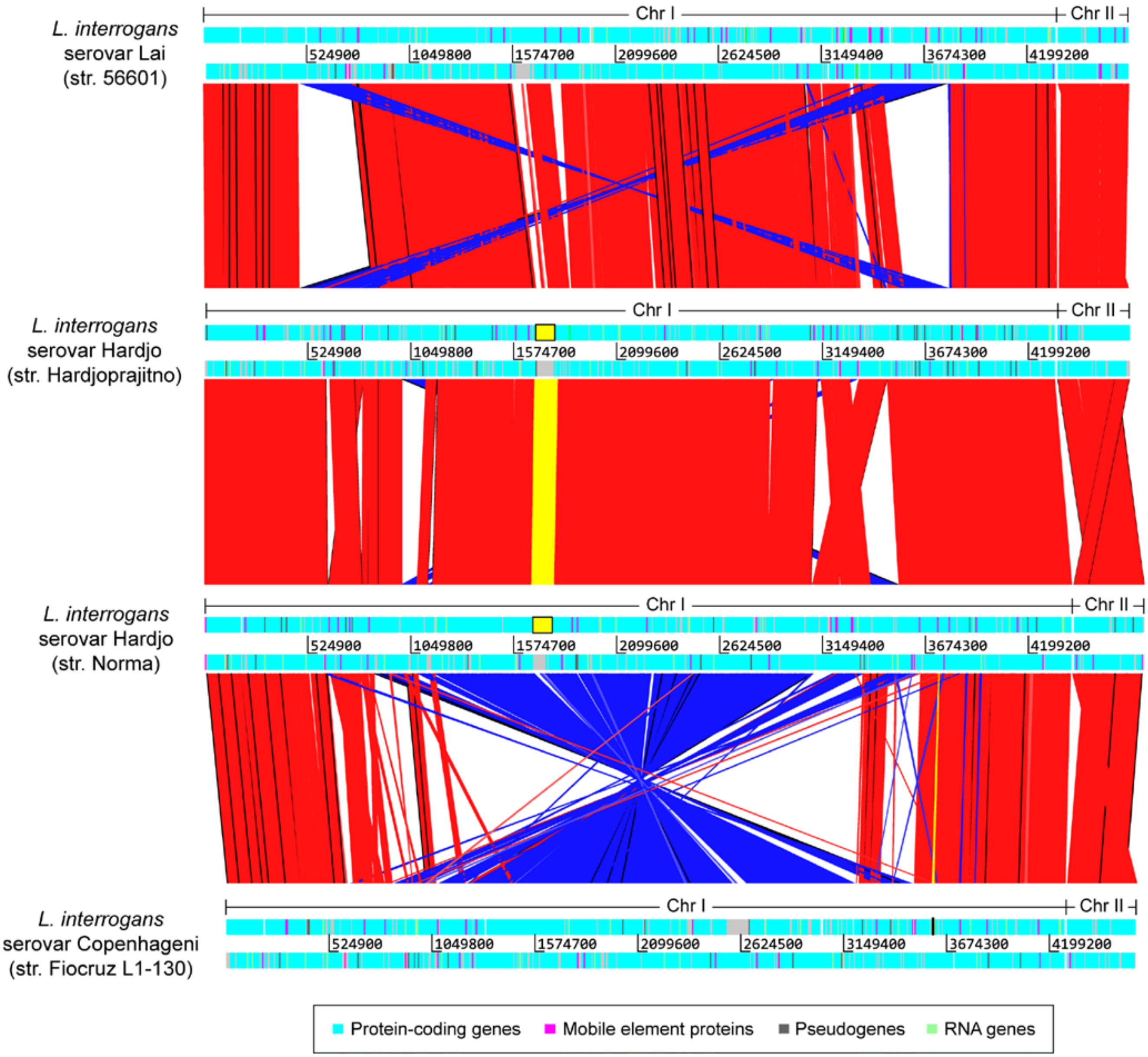

Figure S2. Comparison of the two *L. interrogans* serovar Hardjo genomes included in this study with those of serovars Lai and Copenhageni. Red bands indicate similar regions and blue bands indicate inversions. Sequences corresponding to the rfb loci typical of the Hardjo serovar are highlighted in yellow.
