# Supplementary material for: Whole Genome Sequencing Allows Better Understanding of the Evolutionary History of Leptospira interrogans Serovar Hardjo
Source: PLoS One. 2016 Jul 21;11(7):e0159387. doi: 10.1371/journal.pone.0159387 (PMC4956267; doi:10.1371/journal.pone.0159387)
Supplement: S3 Fig — This tree was built with the amino acid sequences of the acrA genes from the RND transporter gene clusters present in the Leptospira genomes used in this study. The tree was built with PhyML 3.0 using the LG model and 500 bootstrap replicates. Bootstrap values are indicated for branches clustering the genes from different species. Genes from the strain sequenced in this study are indicated in bold. (PDF) [file pone.0159387.s003.pdf]

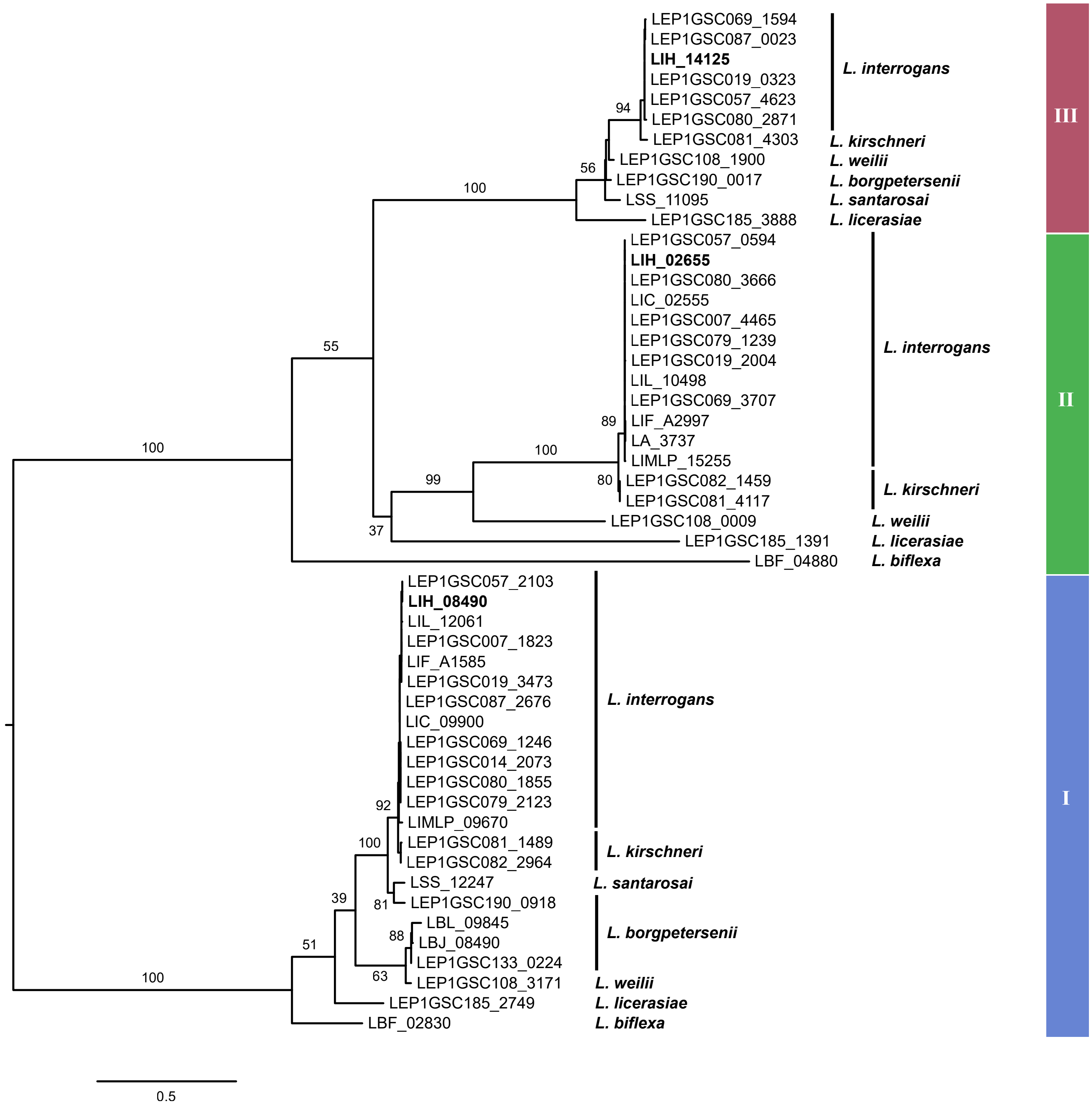

Figure S3. Maximum likelihood tree of acrA genes. This tree was built with the amino acid sequences of the acrA genes from the RND transporter gene clusters present in the *Leptospira* genomes used in this study. The tree was built with PhyML 3.0 using the LG model and 500 bootstrap replicates. Bootstrap values are indicated for branches clustering the genes from different species. Genes from the strain sequenced in this study are indicated in bold.
